# Chapter 1: The Natural History of Intracranial Aneurysms

**DOI:** 10.3390/brainsci16050497

**Published:** 2026-04-30

**Authors:** Paolo Palmisciano, Mario Zuccarello

**Affiliations:** 1 Department of Neurosurgery, University of California, Davis, Sacramento, CA 95817, USA; ppalmisciano@health.ucdavis.com; 2 Department of Neurosurgery, University of Cincinnati College of Medicine, Cincinnati, OH 45219, USA; 3 Division of Pediatric Neurosurgery, Department of Neurosurgery, Cincinnati Children’s Hospital Medical Center, University of Cincinnati College of Medicine, Cincinnati, OH 45229, USA

**Keywords:** intracranial aneurysms, subarachnoid hemorrhage, natural history of brain aneurysms, aneurysm growth, familial intracranial aneurysm, vessel wall imaging

## Abstract

**Highlights:**

**What are the main findings?**

- While intracranial aneurysms affect 2–3% of the adult population, their rupture risk is not constant; it is often highest shortly after formation before the vessel wall potentially stabilizes.
- Aneurysm formation and progression are driven by a complex interplay of hemodynamic stress (wall shear stress), inflammatory vascular remodeling, and genetic susceptibility.

**What are the implications of the main findings?**

- Clinical management is shifting from simple size-based measurements toward individualized models, such as the PHASES score and machine learning, which incorporate morphology, location, and patient-specific factors like smoking and hypertension.
- Longitudinal monitoring now emphasizes detecting growth (more than 1 mm annually) and using technologies like Vessel Wall MRI and AI to identify markers of inflammation and instability.

**Abstract:**

Intracranial aneurysms are common vascular lesions with a highly variable natural history. While most unruptured intracranial aneurysms remain stable throughout life, a biologically aggressive subset progresses to growth and rupture, resulting in aneurysmal subarachnoid hemorrhage with substantial morbidity and mortality. Contemporary evidence demonstrates that aneurysm behavior is dynamic rather than static and reflects the interaction of hemodynamic forces, inflammatory vascular remodeling, genetic susceptibility, and environmental risk factors. Rupture risk is not constant over time and may be highest early after aneurysm formation, followed by a period of relative quiescence in selected lesions. Traditional population-based risk estimates have therefore evolved toward individualized risk stratification incorporating aneurysm size, location, morphology, growth, patient-specific factors, and emerging imaging and computational biomarkers. This chapter reviews the epidemiology, pathobiology, growth patterns, and rupture risk of intracranial aneurysms, integrating foundational observational studies with recent advances in genetics, vessel wall imaging, and predictive modeling. Understanding the natural history of brain aneurysms is essential for balancing the risks of observation against intervention and for guiding future innovations in aneurysm management.

## 1. Introduction

Intracranial aneurysms are focal dilatations of cerebral arteries that arise from progressive weakening of the vessel wall. Their clinical importance derives from the potential for rupture, leading to aneurysmal subarachnoid hemorrhage (SAH), a condition associated with high mortality and long-term neurological disability. With widespread use of noninvasive neuroimaging, unruptured intracranial aneurysms (UIAs) are now detected with increasing frequency, shifting emphasis toward understanding their natural history and identifying lesions at greatest risk for rupture [1].

The natural history of intracranial aneurysms is heterogeneous and dynamic. Rather than static structural abnormalities, aneurysms are thought to evolve over time under the influence of hemodynamic forces, inflammatory processes, genetic susceptibility, and environmental exposures. Contemporary literature emphasizes that rupture risk is not constant but varies based on time and among individuals [2]. This chapter reviews current understanding of aneurysm epidemiology, pathogenesis, growth, and rupture risk, synthesizing classic observational studies with recent advances in genetics, imaging, and risk stratification.

## 2. Epidemiology and Prevalence

Population-based studies estimate the prevalence of intracranial aneurysms at approximately 2–3% in adults, increasing to 3.6–6% in individuals over 50 years of age [3]. In a large-scale Korean cohort study, the standardized incidence of UIAs was found to be 52.2 per 100,000 person-years [4].

Women are affected more frequently than men, with a female-to-male ratio of approximately 3:2, a difference that becomes more pronounced after menopause [1,4]. The risk increases significantly with age; the hazard ratio for individuals aged 60–69 is 15.37 compared to those aged 10–29 [5]. Geographic and ethnic variation is well documented, with higher prevalence and rupture rates reported in Japanese and Finnish populations, suggesting genetic and environmental influences [4,6]. However, some researchers argue that high Finnish rates may be partly due to high medicolegal autopsy rates for sudden deaths (which capture cases often missed in other regions) rather than purely genetic or environmental factors [5,7].

Beyond age and sex, several clinical factors have been studied to independently increase the risk of IA development [3]. Subjects with hypertension have an approximately 1.5-fold higher risk of UIAs. A history of heart disease is associated with a hazard ratio of 2.08. A family history of stroke carries a hazard ratio of 1.77. Smoking and high blood pressure are major contributors to SAH risk. Interestingly, while some studies show high smoking rates among IA patients, others show lower proportions, suggesting regional lifestyle variations [4,6].

Although UIAs are common, aneurysmal SAH is relatively rare, with an annual incidence of approximately 6–10 per 100,000 persons [7]. This disparity suggests a key feature of aneurysm natural history: most aneurysms never rupture. Nonetheless, aneurysmal SAH accounts for up to 85% of spontaneous SAH and carries 30-day mortality rates of 35–45%, with significant cognitive and functional impairment among survivors [1,8].

## 3. Pathogenesis and Aneurysm Formation

The pathogenesis of intracranial aneurysms (IAs) is a complex, multi-step process characterized by the transition of a healthy arterial wall into a pathologically dilated sac. Observational studies suggest that this process is not congenital but likely develops throughout life due to a sophisticated interplay of genetic predisposition, anatomical variations, and modifiable risk factors.

### 3.1. Hemodynamic Stress and Endothelial Dysfunction

The origin of an intracranial aneurysm is rarely a congenital defect; rather, it is considered to be primarily a localized response to the mechanical forces of blood flow [5]. These lesions typically emerge at the bifurcations and major branching points of the Circle of Willis, where the arterial wall must withstand the highest levels of wall shear stress and turbulent flow, and where complex blood flow patterns generate abnormal wall shear stress (WSS). Experimental and computational studies demonstrate that both excessively high and abnormally low WSS contribute to endothelial injury, altered nitric oxide signaling, and maladaptive vascular remodeling [9]. Anatomical variations, such as a hypoplastic segment in the arterial circle, can further exacerbate these hemodynamic insults by forcing blood through narrow or asymmetric channels [10].

Laboratory-based studies suggest that when these physical stresses exceed a certain threshold, they may trigger endothelial dysfunction [11]. The endothelial cells, the thin layer of cells lining the blood vessels, act as mechanical sensors. In response to injury, they begin to release signaling molecules like CCL2 (i.e., Chemokine (C-C motif) ligand 2) [12]. This chemical distress signal initiates a cascade of maladaptive vascular remodeling, characterized by altered nitric oxide signaling and the accumulation of lipids within the vessel wall, effectively setting the stage for structural failure [11,12].

### 3.2. Vascular Wall Remodeling and Inflammation

Once the initial endothelial barrier is compromised, the progression of the aneurysm is thought to be driven by a chronic inflammatory cycle that systematically weakens the arterial wall [11]. A defining histopathologic feature of this stage is the fragmentation of the internal elastic lamina, as evidenced by histopathological studies [13]. Because cerebral arteries lack an external elastic lamina, they are uniquely dependent on this internal layer for structural integrity; its breakdown is the “point of no return” that allows the vessel to bulge. This structural decay is fueled by an influx of macrophages, recruited by the earlier release of CCL2 [12]. These inflammatory cells infiltrate the tunica media and secrete matrix metalloproteinases and pro-inflammatory cytokines, such as IL-1β and TNF-α, which dissolve the extracellular matrix [12,13].

Simultaneously, smooth muscle cells undergo a “phenotypic switch.” Instead of maintaining their healthy, contractile function, they transform into macrophage-like cells that further degrade the matrix, or they undergo apoptosis (programmed cell death), leading to a thinning of the vessel wall [14]. Hence, these experimental studies suggest that the aneurysm’s natural history, whether it remains a stable, incidental finding or progresses towards rupture, depends on the precarious balance between the destructive inflammatory processes and the body’s reparative mechanisms.

### 3.3. Genetic Susceptibility

While environmental stressors like smoking and hypertension are thought to be critical for aneurysm formation and rupture, genetic susceptibility has been observed to be a pivotal part of it [15]. Familial intracranial aneurysms account for approximately 10% of cases and are associated with increased prevalence, earlier presentation, and higher rupture risk [16]. Large-scale genome-wide association studies have identified nearly twenty specific risk loci, such as SOX17 and FGD6, related to vascular development, extracellular matrix integrity, and inflammatory regulation, although no single causative gene has been identified [17].

Beyond common genetic variants, several rare heritable connective tissue disorders provide profound insight into aneurysm pathobiology. Conditions such as Autosomal Dominant Polycystic Kidney Disease, Vascular Ehlers–Danlos Syndrome, and Marfan Syndrome involve mutations in genes responsible for the structural proteins of the blood vessel (e.g., collagen and fibrillin) or the signaling pathways that regulate vessel growth (e.g., TGF-β) [16,18]. In patients with these disorders, the fundamental “scaffolding” of the arterial wall is inherently weaker, leading to a significantly higher prevalence of aneurysms. Furthermore, recent research into somatic mutations, genetic changes that occur in the vessel wall itself during a person’s lifetime, suggests that localized genetic errors in genes like PDGFRB may also play a role in the formation of complex or fusiform aneurysms [19].

## 4. Natural History of Unruptured Intracranial Aneurysms

The natural history of UIAs involves complex patterns of stability, growth, and temporal fluctuations in rupture risk. While many UIAs remain unchanged for years, others undergo morphological evolutions that significantly elevate the danger of subarachnoid hemorrhage.

### 4.1. Stability, Growth, and Morphologic Change

Longitudinal studies demonstrated that most UIAs remain stable over time [20]. However, approximately 5–10% exhibit measurable growth during medium-term follow-up, with reported growth rates of about 7% over 4 years for aneurysms smaller than 8 mm [21]. Aneurysmal enlargement is rarely a linear process; instead, it tends to be episodic, characterized by sudden bursts of expansion that reflect periods of heightened biological activity and inflammation within the aneurysm wall. Identifying such growth is clinically paramount, as it serves as one of the most potent predictors of rupture. Based on population-based and retrospective cohort studies, an annual enlargement of more than 1 mm has been associated with a more than twofold increase in the subsequent risk of rupture [1,20].

Beyond simple size increases, morphologic changes, including irregular shape, lobulation, and development of daughter sacs, further signal a state of instability [11]. These structural changes likely represent focal weaknesses in the wall and uneven remodeling, factors that have been independently linked to a higher risk of hemorrhage [1,10,11].

### 4.2. Temporal Variability of Rupture Risk

A foundational insight from natural history research is that the risk of rupture is not constant but fluctuates throughout the life of the aneurysm. Although difficult to verify directly in humans, some observational and modeling studies suggest that rupture risk may be higher in an early phase after aneurysm formation [22,23]. Based on those studies, during this early phase, the aneurysm wall is thought to be at its weakest before various reparative or “healing” mechanisms can stabilize the lesion [23]. Aneurysms that survive this initial high-risk period may enter a relative “steady state” [24]. In this quiescent phase, growth may be arrested, and the annual risk of rupture decreases significantly. This temporal variability may help explain a common clinical paradox: while most aneurysms that present as active ruptures are small, mostly small, incidentally detected aneurysms remain stable and rarely rupture during a patient’s lifetime [21,24].

## 5. Risk Factors for Aneurysm Rupture

The risk of aneurysm rupture is determined by a complex interplay of anatomical, hemodynamic, and patient-specific factors. While no single metric can definitively predict a hemorrhage, the combination of clinical history and advanced imaging provides a framework for individualized risk stratification.

### 5.1. Aneurysm-Specific Factors

Aneurysm size is the most dominant predictor of rupture, with risk increasing continuously as the diameter expands, particularly beyond 7–8 mm [21,25]. However, size alone is insufficient; its impact is significantly modified by location. Aneurysms located in the posterior circulation (e.g., basilar artery) and the anterior communicating artery have been observed to rupture more frequently and at smaller sizes than those originating from the internal carotid artery or within the anterior circulation [1,25].

Based on pooled cohort data, additional morphological features may further signal aneurysm instability [22,23]. The presence of daughter sacs, blebs, or multiple lobes suggests focal wall weakness [23]. Observational studies report that irregular shape increases rupture risk with an odds ratio (OR) of approximately 4.8 [22]. Similarly, indices such as aspect ratio (aneurysm height divided by neck diameter) and size ratio (aneurysm height divided by parent vessel diameter) have been observed to be strong predictors [1]. Of interest, hemodynamic studies suggest that contact with surrounding structures, such as bone or nerves, can create asymmetrical stress on the aneurysm wall, further increasing instability [16].

### 5.2. Patient-Specific Factors

Individual patient characteristics are thought to play a critical role in the natural history of UIAs. Population studies found that female sex and hypertension are consistently linked to higher rupture rates [1,4,26]. Active cigarette smoking is a major modifiable risk factor, promoting rupture through endothelial dysfunction, chronic inflammation, and oxidative stress [4]. A family history involving first-degree relatives with a history of aneurysmal SAH dramatically increases risk [2,4]. Some observational studies indicate that patients with a familial history face up to a 17-fold increase in rupture risk compared to sporadic cases [16].

### 5.3. Prior Subarachnoid Hemorrhage

A history of SAH from a separate, previously treated aneurysm is one of the strongest predictors of future rupture [27]. These patients often present with multiple aneurysms and experience a heightened period of wall vulnerability in the years immediately following their initial hemorrhage, often necessitating more aggressive surgical or endovascular intervention [1,27].

### 5.4. Emerging Predictive Tools

To manage these variables, clinicians are moving toward multidimensional models that may assist in developing a standardized approach for aneurysm treatment [28,29]. One of the available and most renowned clinical tools, called PHASES score (Population, Hypertension, Age, Size, Earlier SAH, Site), has been developed to estimate 5-year rupture risk [29]. Similarly, computational studies have devised newer machine learning models able to incorporate up to 47 variables, including time-dependent morphological changes (growth rates) and radiomics, to improve risk stratification, particularly for aneurysms smaller than 10 mm [30]. Finally, advanced imaging studies observed that high-resolution MRI may be able to detect contrast enhancement in the aneurysm wall, which may serve as a biomarker for active inflammation and higher risk of potential rupture [31]. Although these tools carry high promises to assist physicians in daily practice, their clinical readiness has yet to be confirmed; further external validation research through multicenter randomized trials is strongly recommended.

## 6. Natural History Across the Lifespan

### 6.1. Age-Related Considerations

The relationship between chronological age and the risk of aneurysm rupture remains a subject of active debate within the neurosurgical community. Some longitudinal data suggest that older individuals experience higher absolute rupture rates, a phenomenon often attributed to the cumulative effects of chronic hypertension and age-related degradation of the vascular wall’s structural integrity [1,23]. However, other researchers argue that these findings may be skewed by treatment selection bias, where healthier, younger patients undergo preventative surgery while older, frailer patients are managed conservatively, as well as competing mortality risks from unrelated cardiovascular diseases [21].

For younger patients, the clinical challenge is distinct. Although they often harbor smaller aneurysms that might initially appear low-risk, they face a significantly higher cumulative lifetime rupture risk due to their longer life expectancy [21,32]. This “time-at-risk” factor may justify a more proactive management strategy, even for small lesions, to prevent a catastrophic event decades in the future [3]. Conversely, in elderly populations, the decision-making process is heavily weighted by increased procedural risks and the presence of comorbidities [8]. In these cases, the potential benefit of intervention must be carefully balanced against the patient’s remaining life expectancy and the inherent risks of surgery or endovascular therapy [1,32].

### 6.2. Multiple Aneurysms

A significant subset of the population, approximately 20% to 30% of patients diagnosed with a UIA, is found to have multiple intracranial aneurysms [25]. This multi-focality is often indicative of a diffuse underlying arteriopathy, suggesting a systemic predisposition to weakened vessel walls driven by genetic factors, smoking, or chronic hemodynamic stress [27]. When multiple aneurysms are present, the risk of rupture is rarely distributed evenly among the lesions. A patient might harbor one stable, thick-walled aneurysm alongside a smaller, irregularly shaped “daughter” aneurysm that poses a much more immediate threat [33]. This clinical reality emphasizes the necessity of a lesion-specific assessment. Clinicians cannot rely solely on a patient-level risk profile; instead, each individual aneurysm must be scrutinized for its specific morphology, location, and hemodynamic environment to determine which, if any, requires prioritized treatment [33]. This nuanced approach ensures that high-risk lesions are addressed while avoiding the unnecessary morbidity of treating stable, low-risk aneurysms.

## 7. Implications for Clinical Management

The clinical management of intracranial aneurysms is defined by a delicate balance between the risks of surgical or endovascular intervention and the natural risk of rupture under observation. Modern management strategies have moved beyond simple diameter measurements to embrace a multidimensional approach to risk stratification and technological integration [32].

Current guidelines emphasize that treatment decisions must be highly individualized, incorporating patient-specific factors such as age, comorbidities, and personal preference alongside aneurysm-specific characteristics like location and morphology [29]. Some population-based tools, such as the PHASES score, have been devised to provide a standardized framework by integrating these variables to estimate 5-year rupture risks and assist clinical management [25]. However, there are currently high-quality external validation studies confirming their accuracy rates, which may limit their clinical utility. Furthermore, population-based tools also have known limitations in capturing the dynamic biological activity of the aneurysm wall, which can lead to the underestimation of risk in certain populations [30,31].

For aneurysms managed conservatively, serial imaging surveillance is the standard of care [32]. The identification of aneurysm growth during follow-up has emerged as a primary actionable risk factor, often triggering a shift from observation to intervention [30]. This surveillance is being revolutionized by Vessel Wall MRI (VW-MRI), which has been studied to detect wall enhancement, serving as a potential surrogate marker for inflammation and wall instability [31]. Deep learning models are also increasingly studied to assist in the detection of small or subtle lesions, with variable, yet very promising, accuracy results to provide automated, morphological calculations [34]. Finally, some AI systems have demonstrated the potential to improve clinician sensitivity in aneurysm detection, rising from 82% to 96% in patient-wise detection, while simultaneously reducing the diagnostic burden on radiologists [1,30]. Although these findings suggest great promise in future management of intracranial aneurysms, they mostly derive from computational studies using retrospective data. Further clinically based studies are highly warranted before standardizing the introduction of radiomics and AI-based tools in clinical practice.

## 8. Future Directions

Despite significant technological leaps, major gaps persist in our understanding of the early life cycle of aneurysms, particularly their initial formation and the kinetics of their growth. Addressing these gaps will require large-scale prospective cohorts that combine standardized imaging with genetic profiling and long-term follow-up [1,2].

The future of aneurysm management lies in the convergence of multiple advanced disciplines. Identifying genetic predispositions will allow for more targeted screening of high-risk individuals [19]. Integrating 4D-MRI and flow dynamics will provide deeper insights into the mechanical stresses that drive wall degradation [31]. By combining AI-driven radiomics with patient-specific genomic and hemodynamic data, clinicians aim to transition from generalized risk scores to precise, personalized predictions of both the risk and the specific timing of potential rupture [30,34].

The ultimate goal of these future directions is to move toward a “biological” rather than “geometrical” understanding of aneurysm stability, ensuring that interventions are reserved for truly vulnerable lesions while minimizing the burden of lifelong surveillance for stable ones.

## 9. Conclusions

The natural history of brain aneurysms is complex, heterogeneous, and dynamic. Epidemiological and observational data suggest that most aneurysms remain stable throughout life, yet a biologically aggressive subset progresses to rupture with devastating consequences. Contemporary histopathological and laboratory-based evidence emphasizes that aneurysm behavior reflects the interaction of genetic predisposition, hemodynamic stress, inflammatory degeneration, and environmental exposures over time. A nuanced understanding of these processes is essential for individualized risk stratification and optimal management.

## Data Availability

No new data were created or analyzed in this study.

## Abbreviations

The following abbreviations are used in this manuscript:

SAH	Subarachnoid Hemorrhage
UIA	Unruptured Intracranial Aneurysms
WSS	Wall Shear Stress

## References

[1] Gibson D.P., Kulwin C.G., Shah K.J., Payner T.D., DeNardo A.J., Amuluru K., Sahlein D.H. (2025). Natural history of cerebral aneurysms: Risk factors for rupture and implications for management. Neuroimaging Clin. N. Am..

[2] Etminan N., Rinkel G.J.E. (2016). Unruptured intracranial aneurysms: Development, rupture and preventive management. Nat. Rev. Neurol..

[3] Vlak M.H.M., Algra A., Brandenburg R., Rinkel G.J.E. (2011). Prevalence of unruptured intracranial aneurysms, with emphasis on sex, age, comorbidity, country, and time period: A systematic review and meta-analysis. Lancet Neurol..

[4] Kim T., Lee H., Ahn S., Kwon O.-K., Bang J.S., Hwang G., Kim J.E., Kang H.-S., Son Y.-J., Cho W.-S. (2016). Incidence and risk factors of intracranial aneurysm: A national cohort study in Korea. Int. J. Stroke.

[5] Brown R.D., Broderick J.P. (2014). Unruptured intracranial aneurysms: Epidemiology, natural history, management options, and familial screening. Lancet Neurol..

[6] Juvela S., Poussa K., Lehto H., Porras M. (2013). Natural history of unruptured intracranial aneurysms: A long-term follow-up study. Stroke.

[7] de Rooij N.K., Linn F.H.H., van der Plas J.A., Algra A., Rinkel G.J.E. (2007). Incidence of subarachnoid haemorrhage: A systematic review with emphasis on region, age, gender and time trends. J. Neurol. Neurosurg. Psychiatry.

[8] Feigin V.L., Rinkel G.J.E., Lawes C.M.M., Algra A., Bennett D.A., van Gijn J., Anderson C.S. (2005). Risk factors for subarachnoid hemorrhage: An updated systematic review of epidemiological studies. Stroke.

[9] Meng H., Tutino V.M., Xiang J., Siddiqui A. (2014). High wall shear stress or low wall shear stress? Complex interactions of hemodynamics with intracranial aneurysm initiation, growth, and rupture. AJNR Am. J. Neuroradiol..

[10] Okahara M., Kiyosue H., Mori H., Tanoue S., Sainou M., Nagatomi H. (2002). Anatomic variations of the cerebral arteries and their embryology: A pictorial review. Eur. Radiol..

[11] Sadasivan C., Fiorella D.J., Woo H.H., Lieber B.B. (2013). Physical factors effecting cerebral aneurysm pathophysiology. Ann. Biomed. Eng..

[12] Aoki T., Nishimura M., Matsuoka T., Yamamoto K., Furuyashiki T., Kataoka H., Kitaoka S., Ishibashi R., Ishibazawa A., Miyamoto S. (2011). PGE2-EP2 signalling in endothelium is activated by haemodynamic stress and induces cerebral aneurysm through an amplifying loop via NF-κB. Br. J. Pharmacol..

[13] Frösen J., Piippo A., Paetau A., Kangasniemi M., Niemelä M., Hernesniemi J., Jääskeläinen J. (2004). Remodeling of saccular cerebral artery aneurysm wall is associated with rupture: Histological analysis of unruptured and ruptured cases. Stroke.

[14] Kanematsu Y., Kanematsu M., Kurihara C., Tada Y., Tsou T.-L., van Rooijen N., Lawton M.T., Young W.L., Liang E.I., Nuki Y. (2011). Critical roles of macrophages in the formation of intracranial aneurysm. Stroke.

[15] Chalouhi N., Hoh B.L., Hasan D. (2013). Review of cerebral aneurysm formation, growth, and rupture. Stroke.

[16] Hitchcock E., Gibson W.T. (2016). A review of the genetics of intracranial berry aneurysms and implications for genetic counseling. J. Genet. Couns..

[17] Yasuno K., Bakırcıoğlu M., Low S.K., Bilgüvar K., Gaál E., Ruigrok Y.M., Niemelä M., Hata A., Bijlenga P., Kasuya H. (2011). Common variant near the endothelin receptor type A gene is associated with intracranial aneurysm risk. Proc. Natl. Acad. Sci. USA.

[18] Xu H.W., Yu S.Q., Mei C.L., Li M.H. (2010). Screening for intracranial aneurysm in autosomal-dominant polycystic kidney disease. Stroke.

[19] Shima Y., Sasagawa S., Ota N., Oyama R., Tanaka M., Kubota-Sakashita M., Kawakami H., Kobayashi M., Takubo N., Ozeki A.N. (2023). Increased PDGFRB and NF-κB signaling caused by highly prevalent somatic mutations in intracranial aneurysms. Sci. Transl. Med..

[20] Boussel L., Rayz V., McCulloch C., Martin A., Acevedo-Bolton G., Lawton M., Higashida R., Smith W.S., Young W.L., Saloner D. (2008). Aneurysm growth occurs at region of low wall shear stress: Patient-specific correlation of hemodynamics and growth in a longitudinal study. Stroke.

[21] Lanzino G., Brown R.D. (2012). Natural history of unruptured intracranial aneurysms. J. Neurosurg..

[22] Greving J.P., Wermer M.J.H., Brown R.D., Morita A., Juvela S., Yonekura M., Ishibashi T., Torner J.C., Nakayama T., E Rinkel G.J. (2014). Development of the PHASES score for prediction of risk of rupture of intracranial aneurysms: A pooled analysis of six prospective cohort studies. Lancet Neurol..

[23] Van Der Kamp L.T., Rinkel G.J., Verbaan D., Berg R.v.D., Vandertop W.P., Murayama Y., Ishibashi T., Lindgren A., Koivisto T., Teo M. (2021). Risk of rupture after intracranial aneurysm growth. JAMA Neurol..

[24] Sonobe M., Yamazaki T., Yonekura M., Kikuchi H. (2010). Small unruptured intracranial aneurysm verification study: SUAVe study. Jpn. Stroke.

[25] Wiebers D.O., Whisnant J.P., Huston J., Meissner I., Brown R.D., Piepgras D.G., Forbes G.S., Thielen K., Nichols D., O’Fallon W.M. (2003). International Study of Unruptured intracranial aneurysms: Natural history, clinical outcome, and risks of surgical and endovascular treatment. Lancet.

[26] Sato K., Yoshimoto Y. (2011). Risk profile of intracranial aneurysms: Rupture rate is not constant after formation. Stroke.

[27] Huhtakangas J., Lehto H., Seppä K., Kivisaari R., Niemelä M., Hernesniemi J., Lehecka M. (2015). Long-term excess mortality after aneurysmal subarachnoid hemorrhage: Patients with multiple aneurysms at risk. Stroke.

[28] Connolly E.S., Rabinstein A.A., Carhuapoma J.R., Derdeyn C.P., Dion J., Higashida R.T., Hoh B.L., Kirkness C.J., Naidech A.M., Ogilvy C.S. (2012). Guidelines for the management of aneurysmal subarachnoid hemorrhage. Stroke.

[29] Fujimura S., Yanagisawa T., Kudo G., Koshiba T., Suzuki M., Takao H., Ishibashi T., Ohwada H., Yamashiro S., Kamphuis M.J. (2025). Development and validation of a prediction model for intracranial aneurysm rupture risk. JAMA Netw. Open.

[30] Sahlein D.H., Gibson D., A Scott J., DeNardo A., Amuluru K., Payner T., Rosenbaum-Halevi D., Kulwin C. (2023). Artificial intelligence aneurysm measurement tool finds growth in all aneurysms that ruptured during conservative management. J. NeuroInterventional Surg..

[31] Larsen N., Von Der Brelie C., Trick D., Riedel C., Lindner T., Madjidyar J., Jansen O., Synowitz M., Flüh C. (2018). Vessel wall enhancement in unruptured intracranial aneurysms: An indicator for higher risk of rupture? High-resolution MR imaging and correlated histologic findings. AJNR Am. J. Neuroradiol..

[32] Loumiotis I., Brown R.D., Vine R., Cloft H.J., Kallmes D.F., Lanzino G. (2011). Small (<10-mm) incidentally found intracranial aneurysms, Part 2: Treatment recommendations, natural history, complications, and short-term outcome in 212 consecutive patients. Neurosurg. Focus.

[33] McDowell M.M., Zhao Y., Kellner C.P., Barton S.M., Sussman E., Claassen J., Ducruet A.F., Connolly E.S. (2018). Demographic and clinical predictors of multiple intracranial aneurysms in patients with subarachnoid hemorrhage. J. Neurosurg..

[34] Liu Q., Jiang P., Jiang Y., Ge H., Li S., Jin H., Li Y. (2019). Prediction of aneurysm stability using a machine learning model based on PyRadiomics-derived morphological features. Stroke.

